# Differential Macrophage Response to Slow- and Fast-Growing Pathogenic Mycobacteria

**DOI:** 10.1155/2014/916521

**Published:** 2014-05-18

**Authors:** A. Cecilia Helguera-Repetto, Rommel Chacon-Salinas, Jorge F. Cerna-Cortes, Sandra Rivera-Gutierrez, Vianney Ortiz-Navarrete, Iris Estrada-Garcia, Jorge A. Gonzalez-y-Merchand

**Affiliations:** ^1^Departamento de Microbiologia, Escuela Nacional de Ciencias Biologicas (ENCB), Instituto Politecnico Nacional (IPN), 11340 México City, DF, Mexico; ^2^Departamento de Inmunobioquímica, Torre de Investigación, Instituto Nacional de Perinatología Isidro Espinosa de los Reyes (INPer), Montes Urales 800, Colonia Lomas de Virreyes, 11000 México City, DF, Mexico; ^3^Departamento de Inmunologia, Escuela Nacional de Ciencias Biologicas, Instituto Politecnico Nacional (IPN), 11340 México City, DF, Mexico; ^4^Departamento of Biomedicina Molecular, Centro de Investigacion y de Estudios Avanzados (CINVESTAV), IPN, 07360 México City, DF, Mexico

## Abstract

Nontuberculous mycobacteria (NTM) have recently been recognized as important species that cause disease even in immunocompetent individuals. The mechanisms that these species use to infect and persist inside macrophages are not well characterised. To gain insight concerning this process we used THP-1 macrophages infected with *M. abscessus*, *M. fortuitum*, *M. celatum*, and *M. tuberculosis*. Our results showed that slow-growing mycobacteria gained entrance into these cells with more efficiency than fast-growing mycobacteria. We have also demonstrated that viable slow-growing *M. celatum* persisted inside macrophages without causing cell damage and without inducing reactive oxygen species (ROS), as *M. tuberculosis* caused. In contrast, fast-growing mycobacteria destroyed the cells and induced high levels of ROS. Additionally, the macrophage cytokine pattern induced by *M. celatum* was different from the one induced by either *M. tuberculosis* or fast-growing mycobacteria. Our results also suggest that, in some cases, the intracellular survival of mycobacteria and the immune response that they induce in macrophages could be related to their growth rate. In addition, the modulation of macrophage cytokine production, caused by *M. celatum*, might be a novel immune-evasion strategy used to survive inside macrophages that is different from the one reported for *M. tuberculosis*.

## 1. Introduction

Tuberculosis remains a global public health problem of enormous scope and proportion, with an estimated one-third of the world's population harbouring a latent infection and approximately 1.5 million deaths occurring annually from the active disease [1]. The unfortunate intersection of the tuberculosis and HIV pandemics in many developing nations has led to enormous morbidity and mortality [2], with the additional recognition of some nontuberculous mycobacteria (NTM) as important etiological agents of disease, aside from that caused by* Mycobacterium tuberculosis* (representative species of the* M. tuberculosis* complex) [3]. The three most common NTM that cause skin and soft tissue infections in humans are* Mycobacterium abscessus*,* Mycobacterium chelonae*, and* Mycobacterium fortuitum* [4], and the most common causes of pulmonary disease are* M. abscessus* and* M. avium *[5]. Some other species have been described to be uncommon causes of human diseases, such as* Mycobacterium branderi*,* Mycobacterium celatum*, and* Mycobacterium bohemicum*. Despite this,* M. celatum* was previously shown to be an underestimated cause of pulmonary and disseminated infections which may even be fatal [6] and has sometimes been misled with* M. tuberculosis* or* M. terrae*-like organisms [7]. We now know that NTM not only cause disease in immunocompromised individuals [3, 8–11], but they also affect the immunocompetent individuals [3, 6, 7, 12].

NTM are widely distributed in the environment with high isolation rates worldwide. These microorganisms can be found in soil and in both natural and treated water sources. Since there is no evidence of animal-to-human or human-to-human transmission of NTM, human disease is suspected to be acquired from environmental exposures. The most common clinical manifestation of NTM is lung disease, but lymphatic, skin/soft tissue, and disseminated diseases are also important [3].

Little is known about the immune response against NTM, but it has been extensively studied for* M. tuberculosis*. Infection with this pathogen is produced by the inhalation of aerosols containing a small number of bacilli [13]. Alveolar macrophages (MΦ) not only represent the first line of defence with their intracellular killing of bacteria and antigen presentation to lymphocytes but also provide the habitat where mycobacteria can reside [14, 15]. By arresting phagosome maturation and phagolysosomal fusion [13],* M. tuberculosis* causes the destruction of the MΦ, which allows the bacilli to infect new MΦ and thus perpetuate the infection [16, 17]. The outcome depends on the resistance of the host and the virulence of the infecting strain [18, 19]. If mycobacteria are not killed in this initial stage, they survive and proliferate inside the macrophages. This intracellular survival is well documented for* M. tuberculosis* [14, 20–24], but it has also been described for a few NTM [14], including* M. marinum* [25] and* M. fortuitum* [26].

Infection of human acute monocytic leukemia THP-1 cells and infection of bone marrow-derived macrophages (BMDM) are widely used models mimicking MΦ-Mtb interactions [15, 27–29]. Through these models, it has been demonstrated that some* M. tuberculosis* subcellular components, such as the cell wall lipoarabinomannan, trigger a cascade of proinflammatory cytokines and chemokines which, in turn, upregulate key components of the host defence against* M. tuberculosis *[15, 18, 28, 30, 31]. Since further analysis is needed to better understand this immune phenomenon between NTM and MΦ, we conducted the present study using different NTM species to elucidate (1) their ability to infect, persist, and proliferate inside their main host cell, the macrophage; (2) their effect on the initial innate immune response of macrophages. We studied two slow-growing mycobacteria,* M. celatum* and* M. tuberculosis*, and two fast-growing mycobacteria,* M. abscessus* and* M. fortuitum*. We report here that intracellular survival of mycobacteria and initial immune response elicited from macrophages might be related to their growth rate. To the best of our knowledge, this is the first evidence of persistence and survival of* M. celatum* inside human macrophages inducing a cell response which is different from that of* M. tuberculosis*, the typical pathogenic slow-growing bacillus.

## 2. Material and Methods

### 2.1. Mycobacterial Culture

*M. tuberculosis* H37Rv,* M. celatum* ATCC 51130 (strain 1908),* M. fortuitum* ATCC 6841, and a clinical isolate of* M. abscessus* (previously identified by rRNA 16S sequencing) were cultured in Dubos medium with 10% of albumin-dextrose-catalase supplement (bovine albumin fraction V 50 g/L, dextrose 20 g/L, and catalase 0.04 g/L) (Becton Dickinson, Franklin Lakes, NJ, USA) and incubated at 37°C. The bacteria were then pelleted by centrifugation and resuspended in RPMI 1640 medium (Gibco, Life Technologies, Grand Island, NY, USA) [17]. Viability was evaluated by counting the colony-forming units (CFUs), and the bacterial suspension was aliquoted and frozen at −70°C.

### 2.2. Cell Line Culture and Infection

The human monocyte THP-1 cell line was used for this study (ATCC TIB 202). The cells were cultured in RPMI 1640 (Gibco) supplemented with 10% heat-inactivated foetal calf serum and 0.45% dextrose (Sigma Aldrich, St. Louis, MO, USA) and incubated at 37°C in 5% CO_2_ incubator to a density of 1 × 10^6^ cells/mL. Cells were counted in a Neubauer chamber after trypan blue staining. THP-1 cells were treated with phorbol-12-myristate-13-acetate (PMA, Sigma Aldrich) at a concentration of 20 nM for 72 h to induce differentiation into macrophage. Mycobacteria were added to the macrophage culture at a multiplicity of infection (MOI) of 5. After 6 h at 37°C and 5% CO_2_, the infected macrophages were washed with Hanks solution 1x (Gibco) to remove extracellular mycobacteria and incubated with fresh medium.

### 2.3. Phagocytosis Rate Evaluation

200,000 macrophages were plated in each well of an 8-well chamber slide (Nunc, Thermo Scientific, Rockford, IL, USA) and infected with the indicated mycobacterial strain (MOI = 5). After 6, 12, 24, 48, and 72 h, cells were fixed with 4% p-formaldehyde, stained with the Kinyoun method (Difco, Becton Dickinson), and analysed by optical microscopy (1000x). The infection/ingestion percentage was determined by counting macrophages with more than one intracellular mycobacterium and macrophages without intracellular mycobacteria. The integrity of the THP-1 macrophage monolayer was evaluated by optical microscopy (1000x; Carl Zeiss Axiostar Plus microscope, Germany). For this purpose, the percentage of integrity was determined by counting macrophages in infected and noninfected cultures (control) at the same time. 100% of integrity corresponded to the macrophage total number in noninfected cultures.

### 2.4. Quantification of Viable Intracellular Mycobacteria

500,000 macrophages were plated in each well of a 24-well flat-bottom plate (Costar, Sigma Aldrich) and infected with the indicated mycobacterial strain (MOI = 5). After 6, 24, and 48 h, the cells were lysed with 0.05% Tween 20 (Sigma Aldrich) and the supernatants were cultured to determine mycobacterial CFUs. In order to investigate if macrophages were responsible for intracellular multiplication control of the mycobacteria tested, CFUs counts obtained from the infected cells were compared to those of the same mycobacteria cultured under the same conditions, but without macrophages. In this latter condition, mycobacteria were grown in RPMI medium because it has been reported previously [32] that this microorganism can grow optimally in this kind of medium.

### 2.5. Production of Reactive Oxygen Species by Macrophages

150,000 macrophages were plated in each well of a 96-well flat-bottom plate (Costar, Sigma Aldrich) and infected with the indicated mycobacterial strain (MOI = 5). 1% Nitro blue tetrazolium chloride (Sigma Aldrich) was added at *t* = 0 after 6 h or 24 h and then incubated for 15 min and the reaction was stopped by adding 10% sodium dodecyl sulphate/0.08 N NaOH; the colour intensity was quantified with a microplate reader (Thermo Multiscan EX, USA) at 600 nm. Cells restimulated with 20 nM PMA were used as our positive control for ROS production (PMA group). Nonstimulated cells (noninfected PMA treated cells) were used as a negative control and considered as our baseline level for ROS.

### 2.6. Production of Cytokines by Macrophages

1 × 10^6^ macrophages were plated in each well of a 24-well flat-bottom plate (Costar, Sigma Aldrich) and infected with the indicated mycobacterial strain (MOI = 5). After 6 and 24 h, the supernatants were collected and analysed by ELISA (TiterZyme, Assay Designs, Ann Arbor, MI, USA) to determine the concentrations of TNF-*α*, IL-1*β*, IL-8, and TGF-*β*, according to manufacturer's protocol.

### 2.7. Statistical Analysis

All the experiments were performed in triplicate and repeated on three independent occasions. The results were analysed by one-way ANOVA with Tukey's post-test, using SigmaStat v.3.1 (Systat Software, San Jose, CA, USA). A *P* < 0.05 was considered statistically significant.

## 3. Results

### 3.1. Slow-Growing Mycobacteria Gain Entrance to THP-1 Macrophages with More Efficiency than Fast-Growing Mycobacteria

**Figure 1:**
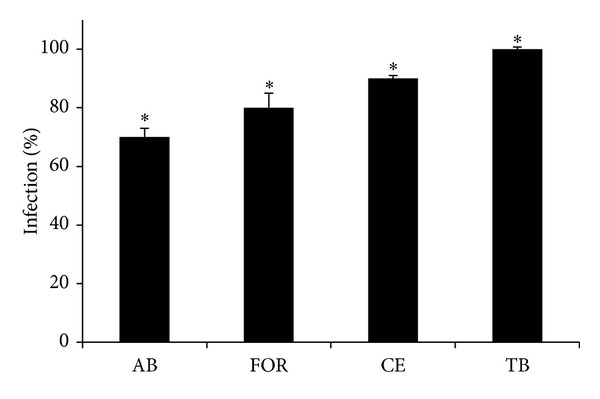
Infection rate of THP-1 cells at 6 h of mycobacterial infections. 2 × 10^5^ THP-1 macrophages were infected with fast-growing (*M. abscessus* and* M. fortuitum*) or slow-growing mycobacteria (*M. celatum* and* M. tuberculosis*) using a MOI of 5. After 6 h of infection, cells were washed and the percentage of macrophages with more than one intracellular mycobacterium was determined. *ν*:* M. abscessus*; *σ*:* M. fortuitum*; *μ*:* M. celatum*; *ο*:* M. tuberculosis*. The experiment was performed in triplicate and repeated on three independent occasions. ANOVA with Tukey's post-test was carried out using a *P* ≤ 0.05. *Significant difference among mycobacteria.

Quantification of intracellular mycobacteria in infected THP-1 macrophages showed that, 6 h after infection, the percentage of infected macrophages was higher in the presence of* M. tuberculosis* (100%) and* M. celatum* (90%), as compared to* M. abscessus* (70%) and* M. fortuitum* (80%) (Figure 1). Therefore, we suggest that macrophages phagocyted the slow growers mycobacteria more quickly than the fast growers. Although significant, we propose that the small differences observed between phagocyted* M. celatum* and fast growers (10% and 20% difference) were also relevant. This is because, although macrophages ingested quite similar quantities of* M. celatum*,* M. fortuitum*, and* M. abscessus*, the cell monolayer was severely compromised throughout the infection of the two fast growers, a fact that was not observed in macrophages infected with* M. celatum* (see next paragraph of results regarding monolayer integrity). Independently of the species of mycobacteria, we observed a 100% rate of infected macrophages at 24 h postinfection (data not shown).

### 3.2. Viable Slow-Growing Mycobacteria Persist inside THP-1 Macrophages

**Figure 2:**
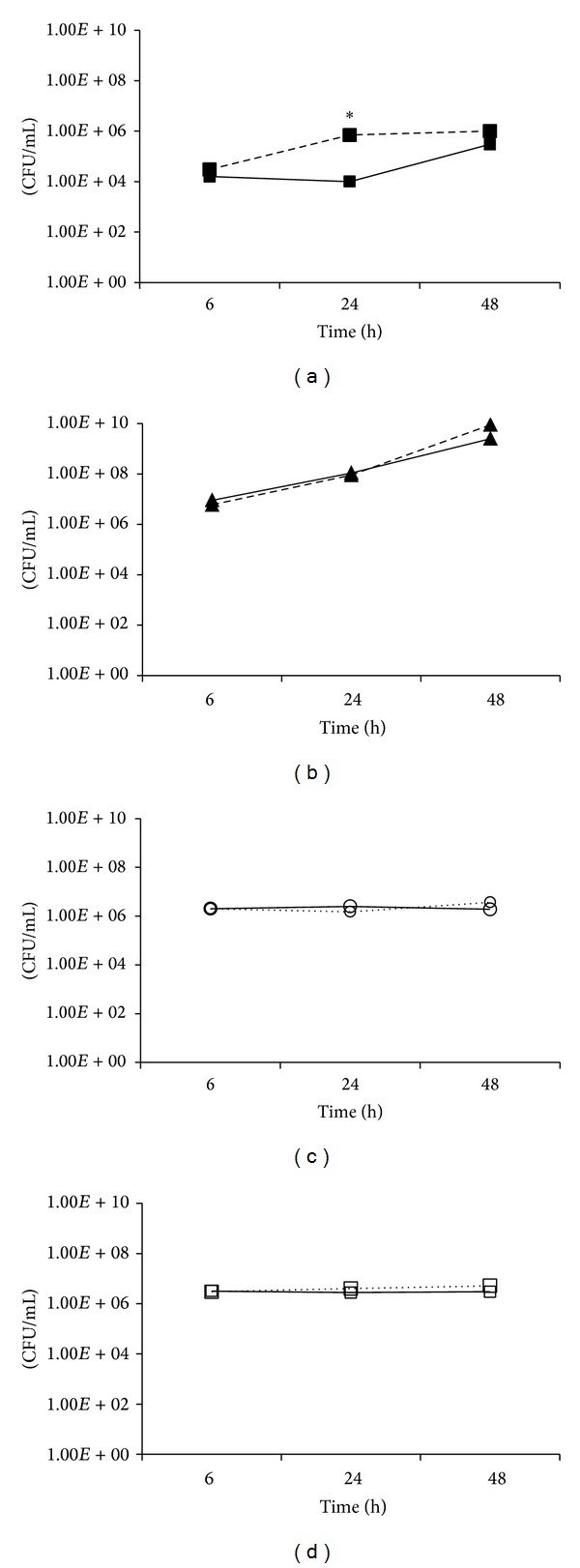
Quantification of viable mycobacteria inside THP-1 macrophages. 5 × 10^5^ THP-1 macrophages were infected with fast-growing (*M. abscessus* and* M. fortuitum*) or slow-growing mycobacteria (*M. celatum* and* M. tuberculosis*) using a MOI of 5. After 6 h of infection, cells were washed and incubated with fresh RPMI medium. Infected cells were incubated for 6 h, 24 h, and 48 h and washed before CFUs determinations (solid lines). As control, each mycobacterium was cultured in medium alone (without macrophages), and the CFUs were determined at the same times (dashed lines). (a)* M. abscessus*; (b)* M. fortuitum*; (c)* M. celatum*; (d)* M. tuberculosis*. The experiment was performed in triplicate and repeated on three independent occasions. ANOVA with Tukey's post-test was carried out using a *P* ≤ 0.05. *Significant differences between the CFUs of mycobacteria that were present inside the macrophages, compared to the CFUs of mycobacteria cultured in medium alone.

In order to demonstrate that intracellular mycobacteria were alive, we performed a viability test (CFUs/ml determination). Our results showed that at least during 48 h, THP-1 macrophages were unable to eliminate any of the mycobacterial species. Regarding fast-growing mycobacteria, there was a 2-log decrease in the CFUs of* M. abscessus* that were present inside the THP-1 macrophages, compared to the CFUs of* M. abscessus* cultured in medium alone (24 h after infection, Figure 2(a)). However, this decrease was no longer evident at 48 h of infection (Figure 2(a)). Although the number of* M. fortuitum* remained the same between those two different conditions (inside macrophages or in a macrophage-free culture), it increased by 2-log from 6 h to 48 h postinfection (Figure 2(b)). In contrast, the numbers of* M. celatum* and* M. tuberculosis* inside the macrophages were similar, and we did not observe any changes in the numbers of these two mycobacteria with the passage of time (Figures 2(c) and 2(d)). The CFUs of these two mycobacteria that were present inside the macrophages were no different from the CFUs of mycobacteria cultured in medium alone, which may indicate that at this MOI (1 : 5)* M. celatum* and* M. tuberculosis* survived but did not proliferate inside macrophages during the first 48 h of infection.

### 3.3. Fast-Growing Mycobacteria Damaged the Monolayer Integrity of THP-1 Macrophages Starting at 48 h of Infection

**Figure 3:**
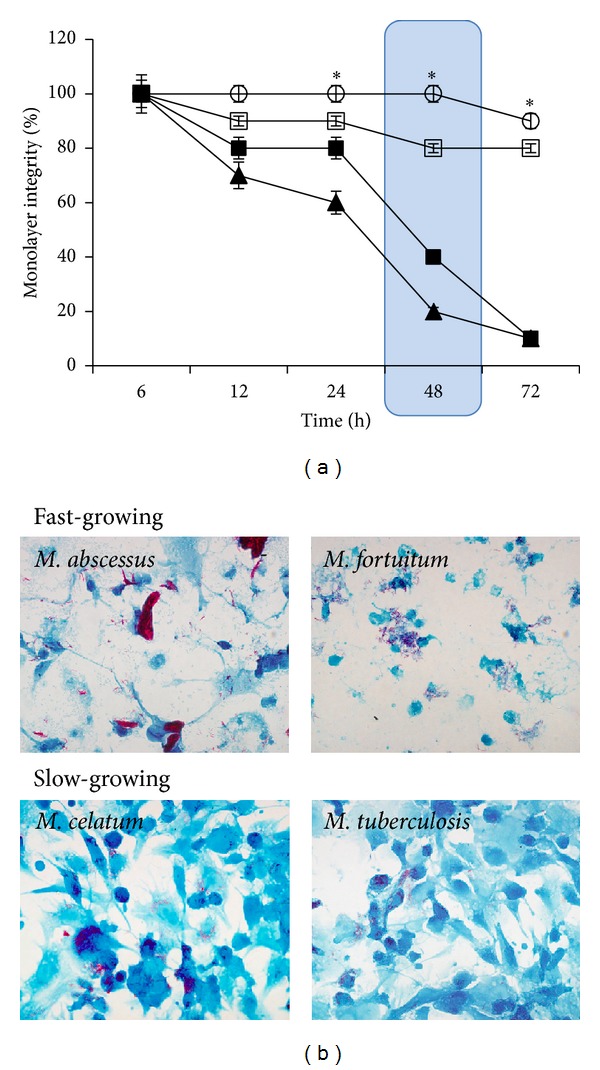
Integrity of THP-1 macrophages monolayer after mycobacterial infection. 2 × 10^5^ THP-1 macrophages were infected with fast-growing (*M. abscessus* and* M. fortuitum*) or slow-growing mycobacteria (*M. celatum* and* M. tuberculosis*) using a MOI of 5. After 6 h of infection, cells were washed and incubated with fresh RPMI medium. Infection kinetics were stopped at 6 h, 12 h, 24 h, 48 h, and 72 h, fixed using 4% p-formaldehyde, and visualized by microscopy. Monolayer integrity of infected cells was compared with monolayer of noninfected macrophages. (a) Monolayer integrity percentage of infected macrophages. Large cell damage is shown within a blue box. *ν*:* M. abscessus*; *σ*:* M. fortuitum*; *μ*:* M. celatum*; *ο*:* M. tuberculosis*. The experiment was performed in triplicate and repeated on three independent occasions. (b) Microscope photographs of THP-1 macrophages at 48 h of infection with the indicated mycobacteria (600x, Kinyoun stain).

The integrity of the THP-1 macrophage monolayer decreased in the presence of fast-growing mycobacteria. Seventy-two hours after infection, just 10% of the monolayer remained intact in the presence of* M. abscessus* and* M. fortuitum*, while 80–90% of the monolayer was intact in the presence of slow-growing mycobacteria (Figure 3(a)). The characteristic cellular morphology of THP-1 macrophages was not altered at 48 h of infection with slow-growing mycobacteria (Figure 3(b)). According to these results, while fast-growing mycobacteria can produce 60 to 80% damage of the macrophage monolayer integrity at 48 h of infection, slow-growing mycobacteria only produced 0 to 20%. Since macrophage monolayer damage at 48 h postinfection produced by fast-growing mycobacteria was too extensive, all of the following experiments were performed within the first 24 h after infection.

### 3.4. Slow-Growing Mycobacteria Do Not Induce the Production of Reactive Oxygen Species (ROS) by THP-1 Macrophages

**Figure 4:**
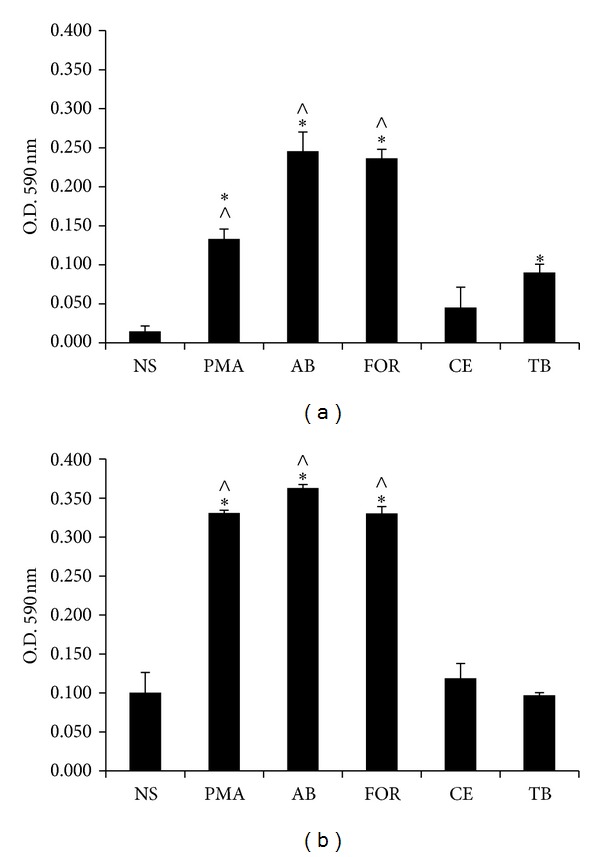
Production of reactive oxygen species by THP-1 macrophages after mycobacterial infection. 1.5 × 10^5^ THP-1 macrophages were infected with fast-growing (AB,* M. abscessus* and FOR,* M. fortuitum*) or slow-growing mycobacteria (CE,* M. celatum* and TB,* M. tuberculosis*), using a MOI of 5. After 6 h of infection, cells were washed and incubated with fresh RPMI medium and 0.1% NBT. Reaction was stopped using 10% SDS in 0.8 N NaOH, and the reduction of NBT was measured at 6 h (a) and 24 h (b). The experiment was performed in triplicate and repeated on three independent occasions. ANOVA with Tukey's post-test was carried out using a *P* ≤ 0.05; NS: nonstimulated cells; PMA: phorbol-12-myristate-13-acetate. *Significant difference compared to nonstimulated cells; ^∧^significant difference compared to* M. tuberculosis*.

Fast-growing mycobacteria induced the production of ROS by THP-1 macrophages at 6 and 24 h postinfection. In contrast, no ROS could be detected with the slow-growing mycobacteria,* M. celatum* and* M. tuberculosis*, even after 24 h of infection (Figure 4). It is probable that the high levels of ROS induced by fast-growing mycobacteria could be associated with the extensive cellular damage that we reported above.

### 3.5. The Cytokine Pattern Induced by* M. celatum* Is Different from the Cytokine Pattern Induced Either by* M. tuberculosis* or by Fast-Growing Mycobacteria

**Figure 5:**
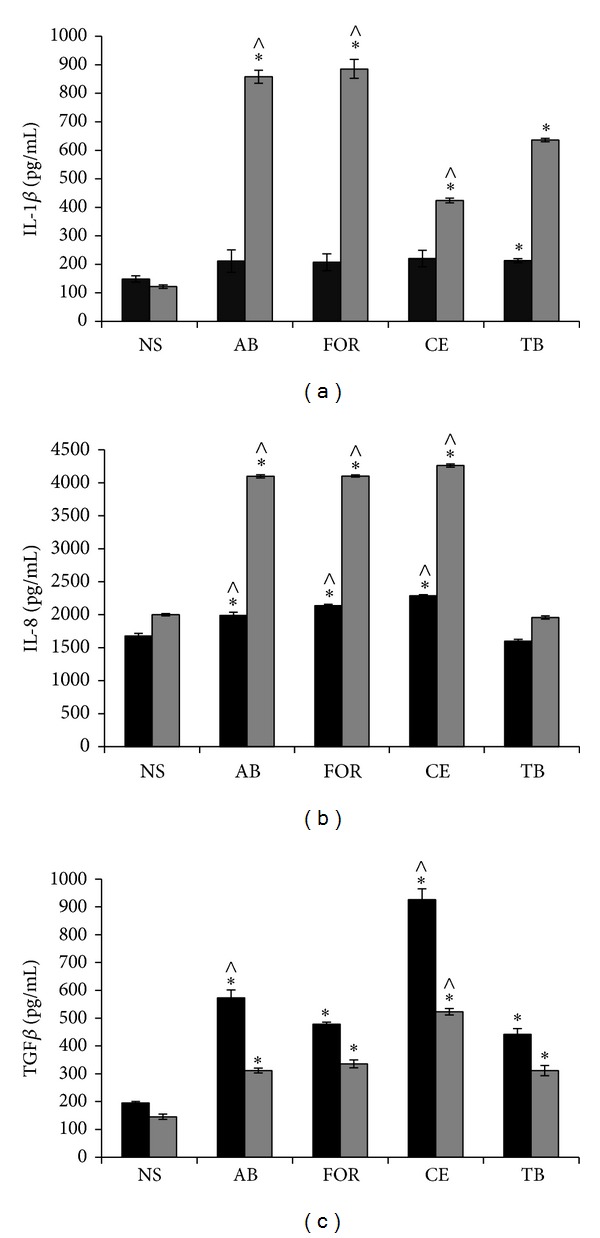
Production of cytokines by THP-1 macrophages after mycobacterial infection. 1 × 10^6^ THP-1 macrophages were infected with fast-growing (AB,* M. abscessus* and FOR,* M. fortuitum*) or slow-growing mycobacteria (CE,* M. celatum* and TB,* M. tuberculosis*), using a MOI of 5. After 6 h of infection, cells were washed and incubated with fresh RPMI medium. Cytokines were quantified at 6 h (black bars) and 24 h (gray bars) after infection. The proinflammatory cytokines IL-1*β* (a) and IL-8 (b) and the anti-inflammatory cytokine TGF-*β* (c) were analysed. The experiment was performed in triplicate and repeated on three independent occasions. ANOVA with Tukey's post-test was carried out using a *P* ≤ 0.05. *Significant differences compared to nonstimulated cells; ^∧^significant differences compared to* M. tuberculosis*. NS: nonstimulated; PMA: phorbol 12-myristate 13-acetate.

Cytokines have an essential role in the modulation of the immune response and, to a large extent, determine the course of a disease [31, 33]. THP-1 macrophages produced no detectable amounts of TNF-*α* at 6 h postinfection in the four tested mycobacteria, but they produced similar amounts of the aforementioned cytokine after 24 h of infection in all studied mycobacteria (approximately 2800 pg/*μ*l; data not shown). IL-1*β* was detected 6 and 24 h after infection; after 6 h no significant differences were observed in any of the mycobacteria, but after 24 h the levels of this cytokine were significantly higher in the presence of fast-growing mycobacteria even though monolayer was compromised (80% integrity at 24 h for* M. abscessus* infections and 60% for* M. fortuitum*). The lowest levels of IL-1*β* were produced in response to* M. celatum* (Figures 5(a) and 5(b)). The fast-growing mycobacteria and* M. celatum* induced the production of high levels of IL-8 at 6 and 24 h postinfection, while no detectable levels of this cytokine were found in the presence of* M. tuberculosis* (Figures 5(a) and 5(b)). At 6 h and 24 h postinfection, the four tested mycobacteria induced the production of the anti-inflammatory cytokine TGF-*β*, with* M. celatum* inducing the highest levels (Figures 5(a) and 5(b)). According to our results we propose that* M. celatum* is a slow-growing mycobacterium with a particular induced cytokine pattern that does not correspond to that produced by a typical slow- (such as* M. tuberculosis*) or fast-growing mycobacterium. We therefore suggest that the immune response caused by* M. celatum* should be further characterized.

## 4. Discussion

The interaction of mycobacteria with their host cell is a complex event, which has not yet been fully characterised, but it has been reported that macrophage receptors bind to molecules on the surface of mycobacteria and trigger phagocytosis. Phagocytosis, as the main process that mediates the entry of mycobacteria into these cells, has been suggested previously [34, 35]. Therefore, we investigated the effect of two fast-growing (*M. abscessus* and* M. fortuitum*) and two slow-growing mycobacteria (*M. celatum* and* M. tuberculosis*) on the response of human macrophages, in order to evaluate whether the growth rate and other particular characteristics of these microorganisms affect their host-pathogen interaction.

Our results showed that* M. tuberculosis* was the species that reached 100% entrance into THP-1 cells at an earlier time (compared to NTM infections), reflecting the high recognition of its surface components by host cells. It has been reported that several mycobacterial envelope lipids are involved in this recognition. These lipids include the family of lipoarabinomannans (LAMs), whose effects on the host innate immune response depend on the chemical modifications of their distal arabinose residues. While* M. tuberculosis* has mannosylated LAMs (ManLAMs),* M. smegmatis*,* M. fortuitum*, and other fast-growing mycobacteria have LAMs with phosphatidylinositol (PiLAMs) [36, 37] and* M. chelonae* has unmodified LAMs [36–38]. This fact might help explain the differences in the phagocytosis process between NTM and* M. tuberculosis*. Even when* M. celatum* entered the host cell more efficiently than* M. fortuitum* and* M. abscessus* at 6 h postinfection, it reached the 100% level of cells infected at the same time as* M. fortuitum*. Hence, we can assume that these two mycobacteria (*M. celatum* and* M. fortuitum*) might have similar LAMs.* M. celatum *surface lipids should be further analysed in order to confirm our hypothesis.

At the same time, we observed a delayed entry of* M. abscessus* into macrophages that might be explained by the fact that this species produces a glycopeptidic biofilm [38], which could mask its surface lipids, resulting in a less phagocytable microorganism. We simultaneously analysed the mycobacterial intracellular viability, the THP-1 monolayer integrity, and the ROS production of those cells. Our results demonstrated that during the first 48 h the slow-growing mycobacteria,* M. celatum* and* M. tuberculosis*, were able to persist inside THP-1 macrophages without causing cellular damage, albeit without proliferating (as has been demonstrated previously for the tubercle bacilli; see [39]), and this could be related to the lack of ROS production in response to these mycobacteria.* M. tuberculosis* actively blocks reactive oxygen species production [16, 29, 40–43], which is related to its immune-evasion strategy ability to persist inside macrophages [44]. It remains to be determined if* M. celatum* blocks reactive oxygen species production by similar mechanisms.

Although both fast-growing mycobacteria were able to proliferate at 48 h postinfection, in an environment almost free of macrophages (10–20%), our results suggest that this phenomenon was due to monolayer destruction. For* M. abscessus*, it has been reported that infection of THP-1 macrophages induces an oxidative environment inside the cell [45] that could explain the decrease in the number of viable intracellular mycobacteria observed at 24 h after infection (ROS related, see Figure 4), when monolayer integrity was still sufficient (80%) to control mycobacterial growth. Although* M. fortuitum* induced a strong ROS response, cells were not able to control the bacterial growth even at 24 h postinfection, at which time we observed significant monolayer reduction (Figure 3(a)). This early macrophage destruction might be due to a known cellular process, such as apoptosis, which has been reported to occur in THP-1 infections with the fast growers* M. smegmatis* and* M. fortuitum*. Macrophage destruction can also be a consequence of a robust production of reactive oxygen species (ROS) in THP-1 cells infected with* M. abscessus* and* M. fortuitum* (Figure 4), as has been reported previously with different host cell/pathogen systems [46–48]. These results confirm the hypothesis that fast-growing mycobacteria induce a very potent immune response when compared to typical pathogenic mycobacteria (such as* M. tuberculosis*) [49].

Macrophages represent the first line of host defense against most bacterial pathogens. Following interaction with the bacteria, macrophages initiate inflammatory responses by secreting cytokines and chemokines [50–52]. Control of* M. tuberculosis* infection is associated with a proinflammatory class, Th1-type cytokine profile [53, 54]. Among the cytokines, two key proinflammatory cytokines for antimicrobial responses are TNF-*α* and IL-1*β* [18, 52, 55]. In our model, TNF-*α* was induced at the same levels by the four tested mycobacteria (*≈*2800 pg/*μ*l), independently of their growth rate and virulence, which reflects the importance of this cytokine in the control of any mycobacterial infection (data not shown).

IL-1*β* is an important cytokine for host immune defense against* M. tuberculosis*, since several studies have demonstrated that IL-1*β*- and IL-1-receptor-knockout mice are more susceptible to* M. tuberculosis* infections than the wild-type animals [56–59]. Our results suggest that (at 6 h postinfection) the secretion of IL-1*β* must be important to control the infection regardless of the mycobacterial species present in the media as this cytokine facilitates the recruitment of other innate immune cells to the site of infection and as it induces the production of IL-8 and mediates proliferation, cellular differentiation, and apoptosis [60, 61]. Cytokine IL-1*β* also seems to be crucial for the inflammasome formation of pathogenic and nonpathogenic mycobacteria [52, 59]. At 24 h postinfection we observed a differential secretion of IL-1*β* among the mycobacteria studied: fast growers induced the highest level. In consequence, the important induction of IL-1*β* secretion by fast-growing mycobacteria is consistent with their ROS production, since both mechanisms contribute to inflammasome activation. This has been suggested for the fast grower* M. abscessus* [62] and has been previously demonstrated only for the slow grower* M. kansasii *[52]. In contrast,* M. celatum* induced the lowest level of IL-1*β* (even lower than that of* M. tuberculosis*), which suggests that this may be another immune-evasion strategy.

The fast-growing mycobacteria and* M. celatum* induced the production of similar levels of IL-8 at both times analysed (6 h and 24 h), while* M. tuberculosis* induced a statistically significantly lower level. IL-8 not only is involved in attracting PMNs (Polymorphonuclears) to the site of infection [63] but also facilitates the elimination of microorganisms by increasing the efficiency of the bactericidal activity of granulocytes [64]. It is also important in the production of an inflammatory response because it is involved in maintaining balance in the granuloma-active infection. In higher concentrations, however, it can help eliminate the mycobacteria by nonoxidative pathways, as has been demonstrated in* M. fortuitum* [64] and* M. smegmatis* [65] infections. The high amounts of IL-8 in the supernatants of fast-growing mycobacteria infections are in concordance with the production of IL-1*β*. However, in the case of* M. celatum*, cytokines other than IL-1*β* might be responsible for inducing the production of IL-8 at 24 h postinfection.

Anti-inflammatory cytokines prevent the cellular damage that can be caused by an excessive Th1-like response. The fast-growing mycobacteria,* M. abscessus* and* M. fortuitum*, induced the production of TGF-*β* at the same levels as in* M. tuberculosis*; but for infections with the fast growers, TGF-*β* production was not enough to prevent cell damage induced by a strong proinflammatory response (as has been demonstrated to occur in* M. tuberculosis* infections).* M. celatum* induced the highest levels of TGF-*β*, which could reduce the innate immune response and favour the persistence of this mycobacterium in the host, despite the proinflammatory response mediated by IL-8 secretion. To the best of our knowledge, this is the first report that suggests the modulation of macrophages cytokine production by* M. celatum*, particularly of IL-1*β* and TGF-*β*, and of the absence of reactive oxygen species during its infection.

## 5. Conclusions

This study provides evidence that growth rate might be related, in some cases, to the intracellular survival of mycobacteria and the immune response that they induce in THP-1 macrophages. Growth rate, however, is not the only determinant of the outcome of the interaction of mycobacteria-macrophages; other factors such as envelope cell lipids and the particular virulence factors of each mycobacterium should be further considered. We suggest that the ability to block reactive oxygen species production by slow-growing mycobacteria is an immune-evasion strategy that putatively promotes their survival and cytokine production in the host, even in NTM species. Finally, our data provides insight into the novel mechanisms that* M. celatum* uses to persist inside its host cell, which should further be characterized in order to gain knowledge about the pathogenic NTM species that cause disease in immunocompetent patients.
